# 

**Published:** 1849-11

**Authors:** 


### CONTENTS

OF NO. V.

---

### ORIGINAL COMMUNICATIONS.

ESSAYS AND CASES.

Art. I.—Practical Medicine and Surgery. By T. S. Bell, M. D., of Louisville, Kentucky, - - 369

REVIEWS.

Art. II.—On the Treatment of Aneurism. By Benjamin W. Dudley, M. D., etc., Professor of Surgery in the Transylvania University, - - - - - - 403

Art. III.—Manual of Physiology. By William Senhouse Kirkes, M. D. Assisted by James Paget, Lecturer on General Anatomy and Physiology, at St. Bartholomew's Hospital, - - - - - - 413

SELECTIONS FROM AMERICAN AND FOREIGN JOURNALS.

Cases of Variola and Vaccine, - - 429

Reflex Nervous Action of the Uterus, - - - - 430

On the Treatment of Pericarditis; especially on the Effects of Bloodletting and Mercury in that Disease, - - 432

Anesthesia from the Local Application of Chloroform, - 435

On Sanguineous Perspiration, - - - - - 436

Phlebotomy in Ancient Times, - - - - - 437

Calomel in Acute Articular Rheumatism, - - - 437

On Large Doses of Sub-Nitrate of Bismuth in Gastro-Intestinal Affections. - - - - - - 438

Clinical Reports of Twenty Cases of Sterility, - - 441

On the Use of Etherial Solution of Gun Cotton in the cure of Erectile Tumors without Operation, - - 448
On the Use of Chloroform in Periodical Neuralgia, - - 449
Arsenic in Cutaneous Diseases, - - - - 451

ORIGINAL INTELLIGENCE.

Medical Department of Louisville University, - - 453
Prof. Dudley's Surgical Papers, - - - - 455
Adulterated Medicines, - - - - - 457
Prof. Miller's Work on Human Parturition, - - 458
Lead Poison, - - - - - 458
Epidemic Dysentery, - - - - 460

